# Exhaled aerosol increases with COVID-19 infection, age, and obesity

**DOI:** 10.1073/pnas.2021830118

**Published:** 2021-02-09

**Authors:** David A. Edwards, Dennis Ausiello, Jonathan Salzman, Tom Devlin, Robert Langer, Brandon J. Beddingfield, Alyssa C. Fears, Lara A. Doyle-Meyers, Rachel K. Redmann, Stephanie Z. Killeen, Nicholas J. Maness, Chad J. Roy

**Affiliations:** ^a^John A. Paulson School of Engineering & Applied Sciences, Harvard University, Cambridge, MA 02138;; ^b^Sensory Cloud, Boston, MA 02142;; ^c^Center for Assessment Technology and Continuous Health (CATCH), Massachusetts General Hospital & Harvard Medical School, Boston, MA 02114;; ^d^Department of Chemical & Biological Engineering, Massachusetts Institute of Technology, Cambridge, MA 02139;; ^e^Division of Microbiology, Tulane National Primate Research Center, Covington, LA 70118;; ^f^Department of Microbiology and Immunology, Tulane University School of Medicine, New Orleans, LA 70118

**Keywords:** aerosols, respiratory medicine, COVID-19, superspreaders

## Abstract

COVID-19 transmits by droplets generated from surfaces of airway mucus during processes of respiration within hosts infected by severe acute respiratory syndrome coronavirus 2 (SARS-CoV-2) virus. We studied respiratory droplet generation and exhalation in human and nonhuman primate subjects with and without COVID-19 infection to explore whether SARS-CoV-2 infection, and other changes in physiological state, translate into observable evolution of numbers and sizes of exhaled respiratory droplets in healthy and diseased subjects. In our observational cohort study of the exhaled breath particles of 194 healthy human subjects, and in our experimental infection study of eight nonhuman primates infected, by aerosol, with SARS-CoV-2, we found that exhaled aerosol particles vary between subjects by three orders of magnitude, with exhaled respiratory droplet number increasing with degree of COVID-19 infection and elevated BMI-years. We observed that 18% of human subjects (35) accounted for 80% of the exhaled bioaerosol of the group (194), reflecting a superspreader distribution of bioaerosol analogous to a classical 20:80 superspreader of infection distribution. These findings suggest that quantitative assessment and control of exhaled aerosol may be critical to slowing the airborne spread of COVID-19 in the absence of an effective and widely disseminated vaccine.

## Significance

Superspreading events have distinguished the COVID-19 pandemic from the early outbreak of the disease. Our studies of exhaled aerosol suggest that a critical factor in these and other transmission events is the propensity of certain individuals to exhale large numbers of small respiratory droplets. Our findings indicate that the capacity of airway lining mucus to resist breakup on breathing varies significantly between individuals, with a trend to increasing with the advance of COVID-19 infection and body mass index multiplied by age (i.e., BMI-years). Understanding the source and variance of respiratory droplet generation, and controlling it via the stabilization of airway lining mucus surfaces, may lead to effective approaches to reducing COVID-19 infection and transmission.

Severe acute respiratory syndrome coronavirus 2 (SARS-CoV-2) transmits through the air by a combination of the large droplets exhaled when people cough or sneeze and the very small droplets people generate in their airways when they naturally breathe (1–4). How exhaled respiratory droplets vary between individuals, evolve over time within individuals, and change with the onset and progression of COVID-19 infection is poorly understood, yet critical to clarifying the nature of COVID-19 transmission—and other highly communicable airborne respiratory diseases, such as influenza and tuberculosis (TB).

Generation of respiratory droplets in exhaled breath can occur by the force of the fast airflows in the upper airways that arise when we breathe, talk, cough, and sneeze. At peak inspiratory flows during normal breathing, air speeds in the trachea and main bronchi can reach turbulent velocities (5). The rush of air over the thin (5 μm to 10 μm) mucus layer lining the airways can break up the mucus surface into small droplets in the way strong winds produce breakup and spray on the surface of the ocean (6). The nature and extent of this droplet breakup is dependent on the surface properties of the mucus itself (6, 7). Among properties most influencing droplet generation and droplet size are surface viscoelasticity (which resists the stretching of mucus surface on breakup) and surface tension (which lowers the energy expended in small droplet creation) (8, 9). In airway lining mucus, both properties vary with lung surfactant type and concentration, as well as with composition and structure of mucus in close proximity to air surfaces (6). Surfactant and mucin compositional and structural changes, driven, in part, by physiological alterations of the human condition—including diet (10), aging (11), and COVID-19 infection itself (12)—may therefore be anticipated to alter droplet generation and droplet size (7) during acts of breathing.

To ascertain whether COVID-19 infection and other phenotypical differences associated with severity of infection risk might alter airborne droplet generation from airway lining fluid during acts of breathing, we conducted two studies in human and nonhuman primates (NHPs). In our first study, we evaluated the exhaled breath of 194 human subjects at two sites to determine exhaled breath particle variations in the human population. In our second study, we measured the exhaled breath from two species of NHPs following experimental infection via inhalation of SARS-CoV-2. We then assessed exhaled breath particle evolution over the time course of exhaled aerosol particles as a function of nasal viral titer. We report on these studies here.

## Results

### Healthy Human Volunteer Study.

**Fig. 1.**
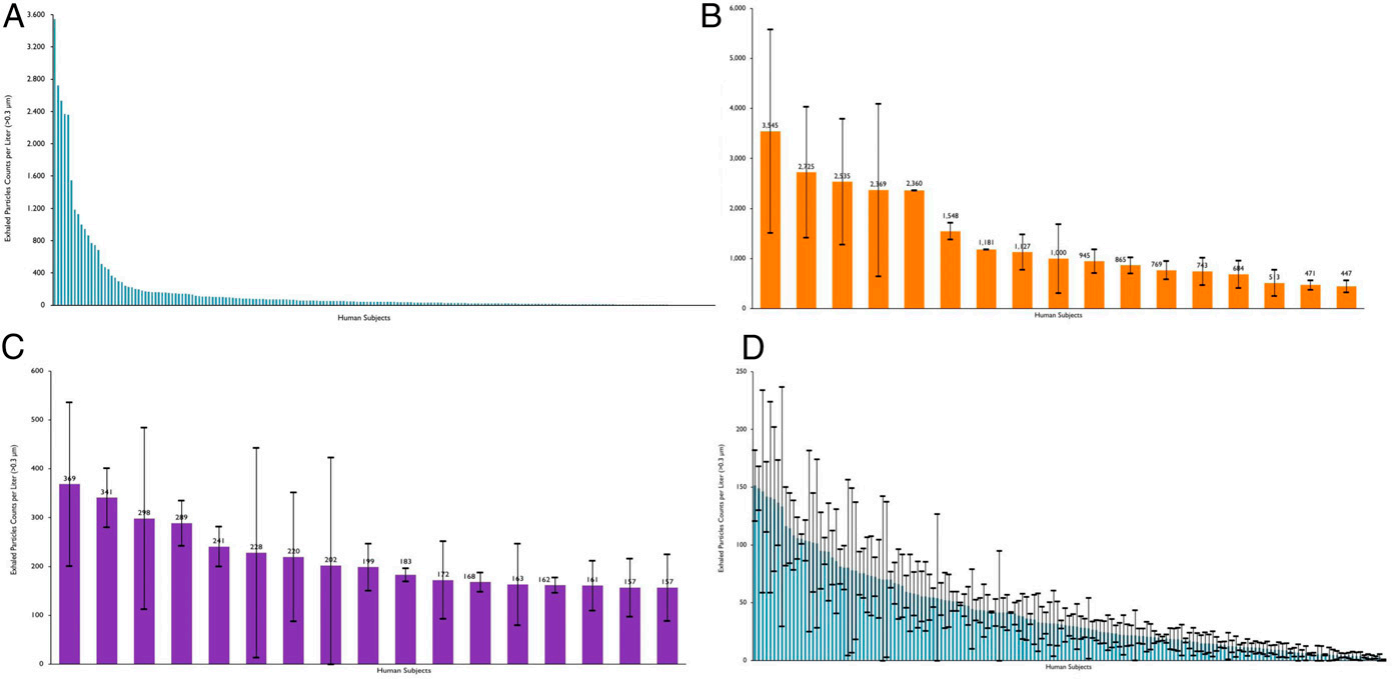
Exhaled breath particles of 74 essential workers at No Evil Foods and of 120 volunteers at Grand Rapids Community College. (*A*) All participants; (*B*) “superspreader” (of aerosol particles) participants (first decile); (*C*) “superspreader” (of aerosol particles) participants (second decile); and (*D*) “low spreader” participants. Data represent particle counts per liter of exhaled air (particle diameter larger than 300 nm) for each of the 194 individuals. Error bars represent SD sample calculations based on 3 to 12 exhaled aerosol count measurements, with each measurement an average of counts over a 5-s time interval.

We evaluated the exhaled aerosol of 194 human volunteers at two sites in North Carolina (74 subjects) and Michigan (120 subjects). We conducted observational cohort studies of essential workers at No Evil Foods in Asheville, NC, and of students, staff, and faculty at Grand Rapids Community College in Grand Rapids, MI, over a total period of 4 d. The results from these measurements are shown in Fig. 1*A*. Exhaled aerosol particle numbers varied by three orders of magnitude between subjects, and were remarkably consistent across the two study sites.

We categorized subjects by those exhaling greater or less than 156 particles per liter of air. We chose this demarcation since the individuals above this threshold aerosol number exhaled 80% of the total particle production from the 194 human volunteers while being less than 20% of the total members of the group—analogous to the conventional definition of superspreading of airborne infectious disease (13). Within this high producing group, we noted that ∼80% of the “superspreader” (of aerosol) production was generated by approximately half of the group, that is, 18 individuals. We qualified as “low spreaders” those 159 individuals who exhaled below 156 particles per liter. The individual data for each category are shown with SDs (Fig. 1 *B*–*D*).

**Fig. 2.**
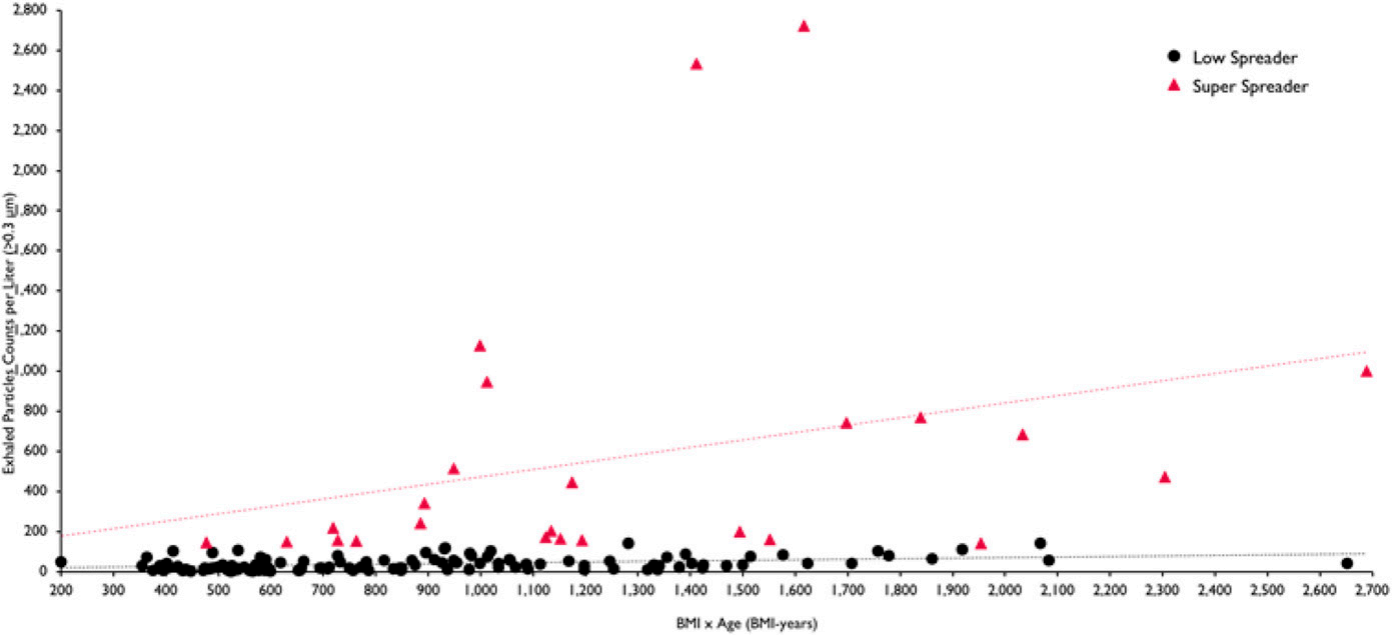
Exhaled breath particles as a function of BMI-years for volunteers reporting age and BMI (*n* = 146). Results of linear regression analysis are shown for the exhaled aerosol numbers from the superspreader and low spreader (of aerosol particles) subjects showing significant correlation, particularly for the superspreader subjects (*r*^2^ = 0.98).

We evaluated relationships between exhaled aerosol particle number and sex, age, and body mass index (BMI). No correlation was found with sex, while significant correlations were observed between exhaled aerosol, age, and BMI—and particularly BMI-years. We characterized each of the 146 individuals for whom we obtained age and BMI information by their age multiplied by their BMI, or by their BMI-years. We noted that half of the group (73 individuals) with lowest BMI-years (less than 650 BMI-years) exhaled significantly less aerosol than the half of the group (73 individuals) with highest BMI-years (above 650 BMI-years) (*P* < 0.015). The BMI-year results are shown in Fig. 2. We note that all volunteers of <26 y of age and all subjects under 22 BMI were low spreaders of exhaled bioaerosol.

### NHP COVID-19 Infection Study.

We studied exhaled aerosol in an NHP model to explore the dependence of exhaled aerosol on COVID-19 infection. We used two NHP species in the study (*Macaca mulatta*, rhesus macaque; and *Chlorocebus aethiops*, African green monkey) to assess interspecies differences in comparison to the human model. We experimentally infected the NHPs (*n* = 8) with SARS-CoV-2 by small-particle aerosol (∼2 µm) and closely monitored them thereafter. Results of mucosal sampling (nasal swabs) showed productive infection in both species, with viral RNA detected as early as +1 d postinfection, viral titers reaching a crescendo in most animals by day +7 postinfection, and clear declination by day +14 postinfection, and undetectable concentrations by study terminus (+28 d postinfection). Corresponding clinical signs of COVID-19 in both species of animals over the course of postinfection observation were considered self-limiting and generally mild. Animals experienced no significant weight loss, a transient, unremarkable fever, and rare lung sounds upon auscultation during clinical examination.

**Fig. 3.**
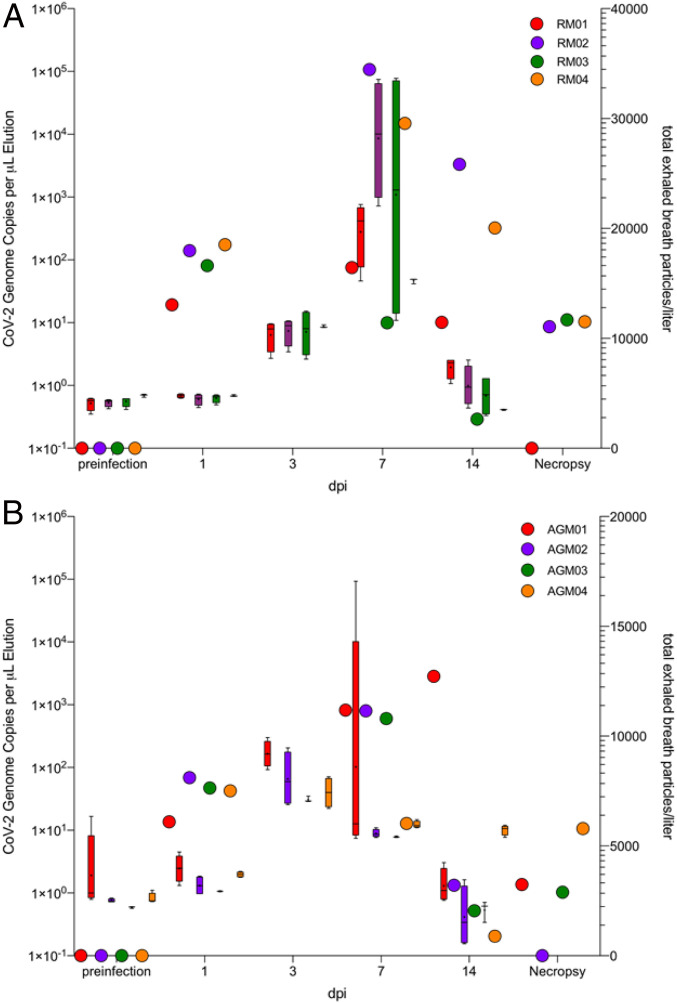
Exhaled breath particles and corresponding genomic SARS-CoV-2 viral RNA in experimentally infected (*A*) rhesus macaques (RM) and (*B*) African green monkeys (AGM). Both groups are segregated by species (*n* = 4; *n* = 8). The corresponding color-matched box-and-whisker plots of total exhaled breath particles represent iterative five 1-min sampling events to genomic viral RNA (color-matched circles) for each animal at each respective time point. Mean calculated correlation between time point-matched exhaled breath particle production and genomic viral RNA showed statistically significant correlations in 75% of the RM (RM01, *r*^2^ = 0.93, *P* < 0.03; RM02, *r*^2^ = 0.99, *P* < 0.004; RM04, *r*^2^ = 0.98, *P* < 0.0008) and 50% of the AGM (AGM02, *r*^2^ = 0.91, *P* < 0.04; AGM03, *r*^2^ = 0.97, *P* < 0.01).

The results of the exhaled breath particle production followed a remarkably similar temporal pattern to that of SARS-CoV-2 viral replication measured in the nasal swabs (Fig. 3). Total exhaled breath particle production began to increase starting at +3 d postinfection and continued to rise by day 7, and decreased to essentially baseline levels by day 14 in both species in all animals across both species.

Although increase of total particles was observed in both species, aerosol particle production increase relative to preinfection totals was more profound in the rhesus macaques (Fig. 3*A*) than in the African green monkeys (Fig. 3*B*), consistent with the sensitivity of rhesus macaques to the development of other active pulmonary infections (14). There was a statistically significant correlation between the production of exhaled breath particles and corresponding nasal viral genomic RNA in three of four of the rhesus macaques (RM01, *r*^2^ = 0.93, *P* < 0.03; RM02, *r*^2^ = 0.99, *P* < 0.004; RM04, *r*^2^ = 0.98, *P* < 0.0008) and two of four of the African green monkeys (AGM02, *r*^2^ = 0.91, *P* < 0.04; AGM03, *r*^2^ = 0.97, *P* < 0.01).

**Fig. 4.**
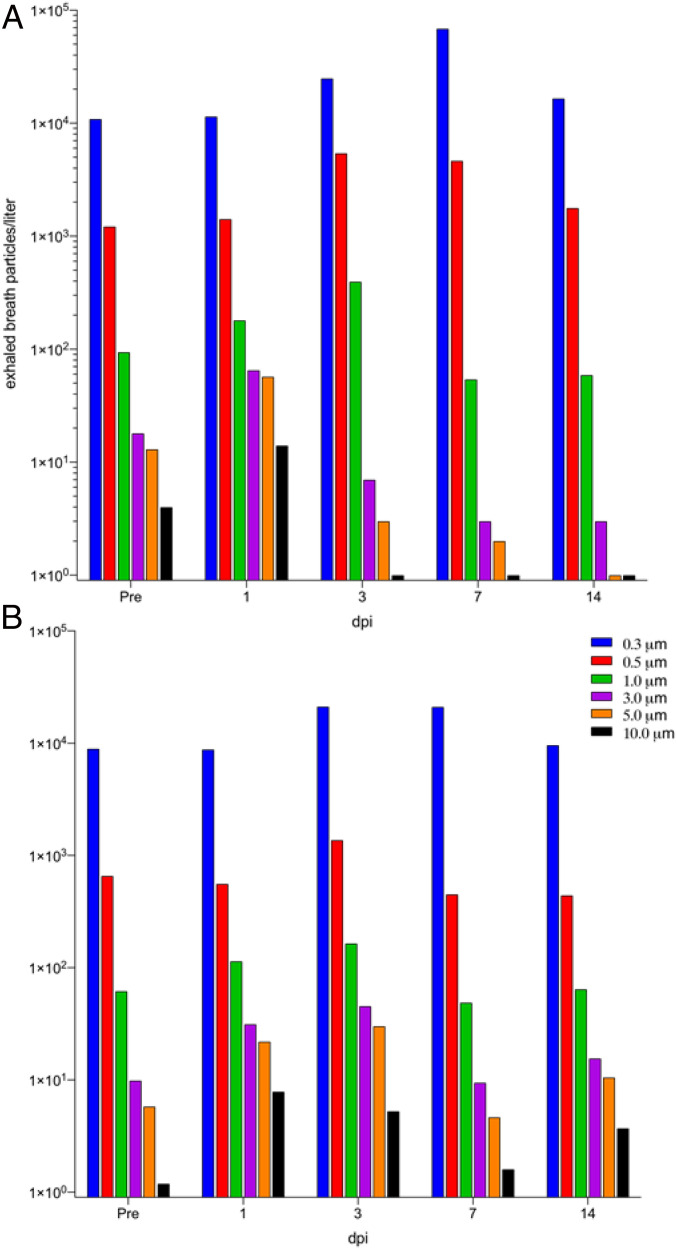
Exhaled breath particles and corresponding particle size distributions in experimentally infected (*A*) rhesus macaques (RM) and (*B*) African green monkeys (AGM); dpi, days postinfection.

The particle distributions of the exhaled breath particles, averaged among species-segregated cohort, changed as COVID-19 disease progressed (Fig. 4). The shift in particle size is typified by a clear increase of particles categorized as <1 µm (collective of the three size bins of 0.3, 0.5, and 1.0 µm) initiating on day +3 postinfection and continuing to trend to a smaller particle size by day 7, with a slight rebound of particle size distribution to baseline by day 14. Collectively, the total number and the relative size distribution of exhaled breath particles produced during experimentally induced COVID-19 is correlated with the viral kinetics of SARS-CoV-2 infection in both the rhesus macaque and African green monkey NHP species.

**Fig. 5.**
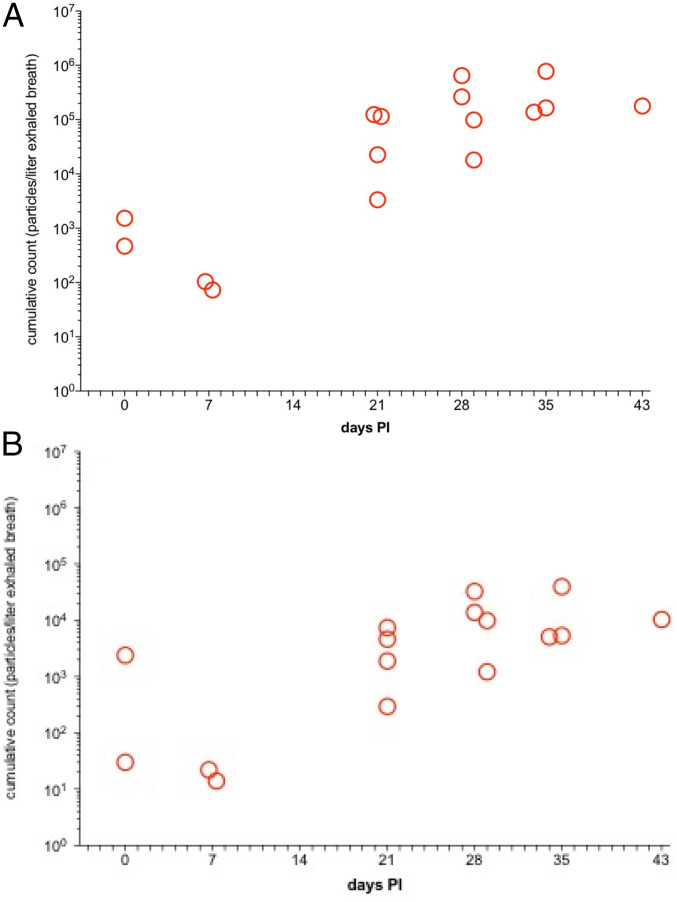
Exhaled breath particles and corresponding particle size distributions in rhesus macaques (*n* = 4) infected with *Mtb*. Total particle counts per liter of air sampled as a measure of production during 10 min of continuous mask sampling for (*A*) all exhaled aerosol particles of >0.5 μm and (*B*) all exhaled aerosol particles of >1.0 μm. The total number of particles increased with time postinfection (PI) (in *A*), with the fraction of particles larger than 1 μm increasing less significantly, reflecting a high submicron fraction (> 90%) from 3 wk PI.

We used a rhesus pulmonary TB model to explore whether changes in exhaled aerosol particle characteristics are observed with lung infections other than COVID-19. TB is an aerosol-acquired pulmonary disease that is caused by exposure to and infection with *Mycobacterium tuberculosis* (*Mtb*). The rhesus macaque represents a highly refined model of clinical TB, with development of active pulmonary disease that shares remarkable similarities with clinical TB in humans (14). Rhesus macaques (*n* = 4) were experimentally infected by small-particle aerosol exposure (∼2 μm) with *Mtb* (Erdman strain) at a target inhaled dose of 400 colony-forming units (CFU). Animals were monitored for development of disease thereafter, including biological outcome parameters correlative with development of disease (e.g., purified protein derivative positivity, C-reactive protein, radiographs, presence of bacilli in lavage fluid). Aerosol parameters measured within the face masks, which also was used in our COVID-19 primate model, included cumulative (total) particle counts and distribution-specific data collection. Results showed animals experimentally aerosol-infected with Mtb strain experienced development of active pulmonary TB over the next several weeks postinfection. Aerosol monitoring of the infected primates via face mask during postexposure observation showed a peak in total particles (∼3.5E+05 particles per liter exhaled breath) at week 4 postinfection (Fig. 5*A*). The distribution of particles collected during infection markedly changed as the disease severity and tempo intensified, with the submicron fraction of particles produced increasing with days postinfection similar to what we observed with COVID-19 infection in the same NHP model (Fig. 5*B*). The temporal development of active pulmonary TB in the infected primate showed an increase of total exhaled breath particles that continued to increase as bacillary load in the lungs of the infected animals increased until experiment terminus, as experimentally induced TB does not resolve without chemotherapeutic intervention. Similarly, total exhaled breath particles increased in the COVID-19−infected primates as viral titers in the mucosal tissues continued to increase. However, due to the self-limiting nature of COVID-19 in the primate model, exhaled breath particles decreased once viral titers began to decrease in the infected animals. These two disease models demonstrate that, although disease pathogenesis differs, the physiological effects of disease induction correlate with the production of increased exhaled breath particles, and, in the case of self-limiting disease (as in the primate model of COVID-19), exhaled breath particle production decreases as disease burden declines.

## Discussion

The generation of respiratory droplets by the breakup of airway lining mucus varies substantially between individuals and with the progression of lung infection. Our findings suggest remarkably similar patterns in two normal human populations in North Carolina and Michigan, and in two kinds of NHP species with two kinds of (viral and bacterial) lung infection. Particularly, that exhaled breath particles in the NHP COVID-19 infection model rise to a crescendo and decrease in size with growth in viral load (Figs. 3 and 4), and vary between healthy humans in close adherence (Fig. 1*A*) with classical superspreading 20:80 rules (13), suggests that the phenomenon of superspreading of COVID-19 (15, 16) may be not only a matter of air currents and proximity of infected and naive hosts but also of phenotype.

Aging (10), diet (11), and lung infection (12) are all known to promote changes in mucus composition and structure. Phenotypical changes in airway lining mucus, such as variation in surfactant composition and subsurface mucin chemistry, are known to alter mucus−air surface propensity to break up into droplets (6, 7), which is consistent with the results shown in Fig. 2.

In a companion article (17), exhaled aerosol particle numbers from a COVID-19−infected human subject were reported between 8 and 12 d postsymptoms. These results were compared with exhaled aerosol particle numbers from three family members in quarantine. As in the NHP infection study reported here (Fig. 3), an extraordinarily high number of exhaled aerosol particles was observed in the infected human subject on days 8 and 9 postsymptoms (2,754 and 1,353 particles per liter), while exhaled particle numbers fell sharply on days 10 and 11 (224 and 29 particles per liter), attaining, in these last days, levels akin to the exhaled aerosol of the other three noninfected family members (in the range of 7 to 198 particles per liter). The present NHP study results (Figs. 3 and 4) shine a fuller light on this phenomenon of respiratory droplet numbers growing with viral load, then falling with the decline in lung infection. Our TB (NHP) results (Fig. 5) indicate that this relationship between increasing exhaled aerosol emission and diminishing droplet size, with the advance of lung infection, might apply to viral and bacterial lung infections other than COVID-19 and TB. While more research needs to be conducted in human and NHP models, it is possible that a transient effervescence in exhaled respiratory droplets may help explain the limited time window post COVID-19 infection during which infected individuals are most contagious.

The strong correlation observed here between advanced BMI-years and greater propensity to generate respiratory droplets (Fig. 2) may be significant in the light of the recognized risk of those with high BMI (18, 19), advanced age (20), or both (21) (the elderly, the obese, and the obese elderly) developing severe symptoms upon COVID-19 infection. Promiscuity of respiratory droplets in the airways heightens the probability that upper airway infection transports deeper into the lungs, promoting severe symptoms, as is observed, with remarkable speed, following intranasal and intratracheal instillation of SARS = CoV-2 in NHPs (22). It also heightens the probability of expelling the aerosol into the environment and transmission of the disease, underlining the transmission risk of living circumstances that bring high-risk (high BMI-year) populations into close proximity for extended periods of time, such as nursing homes.

While those with low BMI-years, including children, appear to be at smallest risk of airway lining mucus breakup and respiratory droplet generation, our NHP results suggest that all individuals, including those with low BMI-years, can be at risk for generating large numbers of respiratory droplets, particularly following lung infection, and therefore argue for the vigilant hygienic protection of the young as well as the old when it comes to the gathering of people within indoor environments where respiratory droplets can linger and accumulate.

Our finding that the proportion of small respiratory droplets [the majority of particles exhaled in all subjects, as has been previously observed (23)] increased at the peak of COVID-19 infection in NHPs (Fig. 4) confirms a previously published observation (17) from the exhaled aerosol profile of a single COVID-19 positive human subject, and suggests that, at peak infection, there may be an elevated risk of the airborne transmission of SARS-CoV-2 by way of the very small droplets that transmit through conventional masks and traverse distances far exceeding the conventional social distance of 2 m.

The scientific response to the COVID-19 pandemic has largely focused on the development of curative drugs and preventive vaccines. In the wait for a cure or an effective widely adopted vaccine, it may be advisable for the scientific community to additionally focus on management of COVID-19 through the restoration of airway lining mucus barrier function, and, notably, in the reduction of the propensity of airway lining mucus to disintegrate under the force of natural breathing, which it is otherwise disinclined to do in the airways of the young and uninfected.

Exhaled aerosol numbers appear to be not only an indicator of disease progression, but a marker of disease risk in noninfected individuals. Monitoring (as a diagnostic) might also be an important strategy to consider in the control of transmission and infection of COVID-19 and other respiratory infectious diseases, including TB and influenza.

## Methods

### Trial Design and Participants.

We conducted observational cohort human volunteer studies in North Carolina and Michigan designed to evaluate exhaled aerosol particle size and number during normal breathing in noninfected humans. In the conducting of the studies and the reporting of our results, we followed Strengthening the Reporting of Observational Studies in Epidemiology statement reporting guidelines. Eligible participants were healthy adults 19 y to 66 y of age, either essential workers at No Evil Foods in Asheville, NC, or students, faculty, staff, and other human volunteers at Grand Rapids Community College in Michigan. Participants were not screened for SARS CoV-2 infection by serology or PCR before enrollment. The trial was conducted on the premises of No Evil Foods and at Grand Rapids Community College. An illustrative (North Carolina) protocol is available in *SI Appendix*. For the North Carolina study, an independent review board (Ethical and Independent Review Services) determined formal Institutional Review Board (IRB) review to be unnecessary when considering the observational nature of the study and the corresponding minimal impact on human subject research. For the Grand Rapids Community College study, the college’s IRB committee approved the protocol.

### Study Procedures.

Participants spent up to 30 min per session while away from work to have their exhaled aerosol particles measured. Exhaled particles were measured by a particle detector (Climet 450-t) designed to count airborne particles in the size range of 0.3 μm to 5 μm. The particle detector was connected to a standard nebulizer tubing and mouthpiece that filters incoming air through a high-efficiency particulate air (HEPA) filter. Each standard nebulizer tubing and mouthpiece was removed from sealed packaging before each subject prior to the subject’s first exhaled particle detection. On subsequent counting maneuvers, the same mouthpiece, tubing, and HEPA filter were replaced into the particle counter system by the participant to insure effective hygiene. Subjects performed normal tidal breathing through a mouthpiece while plugging their noses over 1 to 2 min—beginning with two deep breaths to empty their lungs of environmental particles. Over this time frame, particle counts per liter diminished to a lower baseline number, reflecting particles emitted from breakup of airway lining fluid surfaces in the subject’s airways. Once the lower plateau of particle counts was reached, subjects continued to breathe normally. Three to eight particle counts (average values of particle counts assessed over 6 s) were then averaged to determine the mean exhaled particle count and SD. Participants sat opposite to the study administrator with a Plexiglas barrier separating the participant and the administrator.

### NHP Experimental SARS-CoV-2 Infection.

#### Animals and procedures.

A total of eight male (>7 y of age), purpose-bred rhesus macaques and wild-caught African green monkeys were used in our NHP study of COVID-19; four rhesus macaque monkeys were used in the TB study. NHPs are extremely limited in allocation for the purposes of biomedical research studies, and represent a scarce scientific resource. Therefore, acquisition and use may, at times, trump the balancing of particular desirable characteristics (e.g., gender) in the design of particular experimental cohorts. The use of “all males” in this study was not intentional inasmuch as it was imposed by the process of acquisition and allocation of animals for this study. The African green monkeys species used in a portion of the studies were acquired from a source that does not purpose-breed animals and rather acquires from natural habitat. Accordingly, demographics on these animals are limited (e.g., age) and are only estimated from dental record. Animals were exposed to SARS-CoV-2 (BEI, USA-WA1/2020, NR-52281) and *Mtb* (Erdman) by small-particle aerosol previously characterized in our laboratory inhalation system (24). Animals received (for the COVID-19 studies) an inhaled dose of ∼2.5 × 10^3^ tissue culture infectious dose 50 (*SI Appendix*) and (for the TB studies) an inhaled dose of 466 ± 237 CFU. Animals were observed for 28 d or 60 d postinfection (COVID-19 or TB studies, respectively) including twice daily monitoring by veterinary staff. In our COVID-19 studies, mucosal and other biosamples were collected at 7 d before infection, at days 1, 3, 7, 14, and at necropsy (day 28) after infection. In our TB studies, mucosal and other biosamples were collected at 7 d before infection, at days 1, 7, 14, 21, 28, 35, 42, and at necropsy. During biosampling events and physical examination while anesthetized and in dorsal recumbency, and experiencing normal respiration, animals were individually sampled for exhaled breath aerosols. This sampling was performed using a modified pediatric face mask fitted with a HEPA-filtered inspiration port, and a corresponding sampler for exhalation. A particle counter (Thermo Systems Inc. AeroTrak handheld particle counter Model 9306-V2) was used to sample exhaled breath particles for five 1-min intervals at every sampling time point. Exhaled breath particle data were collected in a cumulative fashion.

#### Quantification of swab viral RNA.

Nasal swabs were collected in 200 µL of DNA/RNA Shield and extracted for viral RNA using the Quick-RNA Viral kit. Samples were then quantified using RT-qPCR (*SI Appendix*, *Methods*).

#### Ethics.

The Institutional Animal Care and Use Committee of Tulane University reviewed and approved all the procedures for this experiment. The Tulane National Primate Research Center is fully accredited by the Association for Assessment and Accreditation of Laboratory Animal Care. All animals are cared for in accordance with *Guide for the Care and Use of Laboratory Animals* (25). The Tulane Institutional Biosafety Committee approved the procedures for sample handling, inactivation, and removal from biosafety level 3 (BSL-3) containment.

## Supplementary Material

Supplementary File

## Data Availability

All data from this study are presented in the article and *SI Appendix*.
